# Investigation of carbapenem-hydrolysing Klebsiella oxytoca strains lacking detectable carbapenemase genes

**DOI:** 10.1099/jmm.0.002139

**Published:** 2026-03-13

**Authors:** Hiroshi Teraguchi, Ayaka Oda, Satoyo Wakai, Sayoko Kawakami, Koki Yoshida, Wataru Hayashi, Norikazu Kitamura, Yo Sugawara, Naoya Ohara, Motoyuki Sugai, Koji Yahara, Aki Hirabayashi

**Affiliations:** 1 Department of Clinical Laboratory, Kindai University Nara Hospital, Nara 630-0293, Japan; 2 Dental School, Okayama University, Okayama 700-8558, Japan; 3 Antimicrobial Resistance Research Center, National Institute of Infectious Diseases, Japan Institute for Health Security, Tokyo 189-0002, Japan; 4 Department of Oral Microbiology, Graduate School of Medicine, Dentistry and Pharmaceutical Sciences, Okayama University, Okayama 700-8558, Japan

**Keywords:** *bla*_OXY-2_, clavulanic acid, *Klebsiella oxytoca*, MIC, modified carbapenem inactivation method

## Abstract

**Introduction.** Carbapenemase-producing *Enterobacterales* (CPE) pose a clinical concern due to limited treatment options and plasmid-mediated spread of carbapenemase genes. The modified carbapenem inactivation method (mCIM) is increasingly used in clinical settings to detect CPE.

**Gap Statement.** Interpretation of mCIM results for isolates with reduced carbapenem susceptibility remains challenging.

**Aim.** To assess the interpretation of positive mCIM results in *Klebsiella oxytoca* isolates lacking detectable carbapenemase genes and exhibiting reduced carbapenem susceptibility and to propose a phenotypic testing strategy for accurate evaluation.

**Methodology.** Antimicrobial susceptibility testing with clavulanic or dipicolinic acid supplementation, phenotypic carbapenemase assays, carbapenem hydrolysis assays and whole-genome sequencing (WGS) were conducted on *K. oxytoca* strains.

**Results.** *K. oxytoca* strains were isolated from both blood and urine samples of a patient in a Japanese hospital. Although classified as susceptible by clinical breakpoints, these isolates exhibited reduced susceptibility to carbapenems. Although these strains were mCIM-positive, the zinc-supplemented carbapenem inactivation method did not support the presence of metallo-*β*-lactamase (MBL) activity. MIC reductions in the presence of CVA were consistent with inhibition of a class A *β*-lactamase. In contrast, dipicolinic acid supplementation did not reduce the meropenem MIC, ruling out MBL production. Furthermore, WGS revealed that both *K. oxytoca* strains lacked known carbapenemase genes, with *bla*_OXY-2-4_ being the only identifiable *β*-lactamase gene encoding a class A enzyme. No novel *β*-lactamase genes were identified; however, an enhanced *bla*_OXY-2-4_ expression-associated point mutation was found in the promoter region. Both *K. oxytoca* strains exhibited carbapenem-hydrolysing activity, although to a lesser extent than that of the MBL-producing strain.

**Conclusion.** We identified *K. oxytoca* strains that lacked known carbapenemase genes but that yielded positive mCIM results, possibly due to OXY-2 overproduction. Our study findings emphasize the need for cautious mCIM result interpretation for *K. oxytoca* and highlight the diagnostic value of combined susceptibility testing with clavulanic and dipicolinic acids to distinguish the underlying resistance mechanisms. Combining complementary clinical laboratory tests will enable clarification of the factors underlying mCIM positivity and guide clinical decision-making.

## Introduction

Antimicrobial-resistant bacteria represent a global health concern, with particular emphasis on the increasing prevalence of carbapenem-resistant organisms, with limited treatment options. Accordingly, the World Health Organization (WHO) has classified carbapenem-resistant *Enterobacterales* (CRE) as a critical-priority pathogen [1]. Carbapenem resistance in CRE arises mainly through carbapenemase production, porin channel loss or mutation and efflux pumps. Among these, carbapenemase-producing *Enterobacterales* (CPE) are of particular clinical importance, as they exhibit resistance to broad spectrum antimicrobials, including penicillin, cephalosporin and carbapenem. They often harbour multiple resistance genes, thus frequently displaying multidrug resistance and complicating effective treatment. Notably, the resistance genes in CPE are commonly located on plasmids that facilitate horizontal gene transfer to other bacterial species. Therefore, rapid and accurate screening for CPE is critical for appropriate antimicrobial selection and implementation of infection-control measures in clinical settings.

According to the Clinical and Laboratory Standards Institute (CLSI), *Enterobacterales* with a meropenem (MEM) MIC of ≤1 µg ml^−1^ are considered susceptible [2], whereas the European Committee on Antimicrobial Susceptibility Testing (EUCAST) defines susceptibility at an MIC of ≤2 µg ml^−1^ [3]. However, EUCAST recommends carbapenemase screening for isolates with a MEM MIC >0.125 µg ml^−1^, setting this value as the epidemiological cutoff for screening purposes. Recently, our national genomic surveillance conducted whole-genome sequencing (WGS) of all *Enterobacterales* isolates that met the epidemiological cutoff values [4]. This analysis identified dozens of isolates carrying carbapenemase genes that remained undetected by routine antimicrobial susceptibility testing [4], highlighting the necessity of including strains with low MICs into surveillance to effectively monitor the prevalence and spread of carbapenemase genes. In local hospitals and clinical laboratories where genome sequencing is not routinely performed, several inexpensive and rapid phenotypic tests [e.g. the modified carbapenem inactivation method (mCIM) [5] and Carba NP test [6]] are vital in routine carbapenemase production detection and differentiation. mCIM is endorsed by CLSI, and previous comparative studies [5] have demonstrated that it currently provides optimal performance among the available methods.

However, to date, no studies have evaluated mCIM sensitivity and specificity for isolates with reduced MEM susceptibility (MIC=0.25, 0.5 or 1 µg ml^−1^), classified as susceptible according to CLSI breakpoints, following the confirmation of resistance gene presence by WGS. In clinical settings, interpreting mCIM results for such borderline isolates remains challenging. In this study, we focused on *Klebsiella oxytoca* isolates with reduced carbapenem susceptibility but positive mCIM results, identified from the blood and urine cultures of hospitalized patients in a Japanese medical facility. *K. oxytoca* typically harbours a chromosomally encoded OXY-type *β*-lactamase gene (*bla*_OXY_), conferring intrinsic resistance to certain cephalosporins. The OXY-type *β*-lactamase belongs to class A *β*-lactamases and is overproduced due to mutations in its promoter region [7]. A carbapenem-resistant isolate (MEM MIC ≥4 µg ml^−1^) has been associated with OXY-type *β*-lactamase overproduction, although it tested mCIM-negative, unlike the strains in this study [7].

In this study, we conducted detailed antimicrobial susceptibility testing, *β*-lactamase activity assays and WGS of two *K. oxytoca* isolates. Based on these analyses, we propose a phenotypic testing strategy for these isolates to improve the interpretation of carbapenemase detection.

## Methods

### Identification and genome sequence analysis of *K. oxytoca* isolates with low carbapenem susceptibility

Two *K. oxytoca* strains (JBBDAAF-23-N014 and JBBDAAF-23-N015) were isolated from the blood and urine samples of a patient in a Japanese medical facility. At the medical facility, these two strains exhibited reduced carbapenem susceptibility but were still classified as susceptible according to CLSI breakpoints and tested mCIM-positive. The strains were sent to the National Institute of Infectious Diseases (NIID) for further analysis and subjected to WGS using Illumina NovaSeq platforms, followed by a search for *β*-lactamase genes, as described previously [4]. An in-house Perl script was used to extract the nucleotide sequence of the *bla*_OXY-2-4_ promoter region using blastn and to detect point mutations. Hidden Markov model (HMM) profiles of *β*-lactamases [8] were also used to search for potentially novel *β*-lactamases using the HMM search algorithm implemented in the HMMER program [9]. The raw short-read data for the two strains were deposited in the DDBJ under the BioProject accession no. PRJDB20593.

### Antimicrobial susceptibility testing

An E-test was performed to determine the MICs of MEM and imipenem (IPM) according to the manufacturer’s instructions (Sysmex bioMérieux, Tokyo, Japan) at the NIID. The MICs of antimicrobial agents were determined using the broth microdilution method. IPM, biapenem (BPM), doripenem, faropenem and tebipenem (TBP) were tested using the dry plate system, Eiken (Eiken Chemical Co., Ltd.; NQJ2 order panel). In addition, to evaluate the inhibitory effect of clavulanic acid (CVA), the MICs of the above-mentioned antimicrobial agents were determined in the presence of CVA at a final concentration of 4 µg ml^−1^. For MEM, the broth microdilution method was performed using manually prepared dilutions, rather than Eiken dry plates (see Fig. S2). Furthermore, to assess the inhibitory effect of dipicolinic acid (DPA) on MEM, MIC testing was performed by the broth microdilution method with DPA supplementation to MEM at a final concentration of 100 µg ml^−1^. Categorization was performed according to the CLSI guideline MIC breakpoints: susceptible (S), intermediate (I) or resistant (R) [2].

### Carbapenemase-detecting phenotypic tests

mCIM, EDTA-carbapenem inactivation method (eCIM) and zinc-supplemented carbapenem inactivation method (zCIM) tests were conducted on two *K. oxytoca* isolates as previously described [10,11]. The CLSI guidelines [2] were used to identify carbapenemases. The mCIM, eCIM and zCIM results were interpreted according to the CLSI guidelines [2] and a previous report [11]. The MASTDISCS *combi Carba plus* disc system D73C (MAST-*Carba plus*; Mast Group, Bootle, Merseyside, UK) was used to detect carbapenemases as per the manufacturer’s instructions [12]. Metallo-*β*-lactamase (MBL): B−A≥5 mm and |C−A| and |D−A|<5 mm; KPC: C−A≥5 mm and |B−A| and |D−A|<5 mm; OXA-48: no synergy observed on A−B, C or D and E≤10 mm; AmpC with porin loss: C−A≥5 mm and D−A≥5 mm and |B−A|<4 mm; negative: differences among A, B, C and D all ≤2 mm and E>10 mm.

### Carbapenem-hydrolysing activity quantification

Carbapenem-hydrolysing activity quantification was performed as described previously [13] with modifications. Briefly, after adjusting the bacterial suspension to an OD of 0.7 at 600 nm, 50 µl of the twofold-diluted suspension was mixed with 50 µl of PBS with or without IPM (final concentration of 50 µg ml^−1^) in an ultraviolet-transparent 96-well plate. The absorbance at 297 nm was measured at 5, 30, 60, 120, 180, 240 and 300 min using a VICTOR Nivo microplate reader (Revvity, MA, USA). Experiments were conducted in triplicate and the mean values were calculated. To calculate the net IPM absorbance, the background absorbance was subtracted from the gross absorbance at 297 nm, i.e. subtracting the absorbance of the bacterial suspension without IPM from that of the bacterial suspension with IPM. The per cent hydrolysis was calculated as described previously [13] and plotted over time. *K. oxytoca* strain ATCC 13182 and *Escherichia coli* strain MS5260 carrying the *bla*_NDM-1_-positive plasmid pNDM-HU01 (accession no. AB769140) were used as the representative non-CPE and CPE strains, respectively.

## Results

Both the JBBDAAF-23-N014 and JBBDAAF-23-N015 strains exhibited an MIC of 0.125 µg ml^−1^ for MEM and 0.25 µg ml^−1^ for IPM, as determined by the E-test conducted at the reference laboratory (Fig. S1, available in the online Supplementary Material). These results were mostly consistent with routine antimicrobial susceptibility testing performed at the original hospital laboratory, revealing MICs of 0.125 µg ml^−1^ for MEM and either 0.5 or 0.25 µg ml^−1^ for IPM (Table S1). Table S1 presents the MICs of 18 antimicrobial agents (including MEM and IPM), determined at the hospital laboratory. For example, the MICs for aztreonam and ceftriaxone were ≥64 and ≥128 µg ml^−1^ for piperacillin/tazobactam, ≤1 µg ml^−1^ for cefmetazole and either 2 or 4 µg ml^−1^ for cefepime.

Table 1 presents the results of the antimicrobial susceptibility testing with and without CVA, an inhibitor of class A *β*-lactamases. CVA supplementation reduced the MIC values of carbapenems as follows: in strain JBBDAAF-23-N014, the MIC of IPM decreased from 0.5 to 0.12 µg ml^−1^. In strain JBBDAAF-23-N015, the MICs of MEM, BPM and TBP were reduced from 0.12, 0.5 and 0.25 µg ml^−1^ to 0.06, 0.25 and≤0.06 µg ml^−1^, respectively. Subsequently, we performed the MIC testing of MEM using the broth microdilution method across a wide concentration range (i.e. 0.007–16 µg ml^−1^), accompanied by inhibition assays with DPA, an inhibitor of class B carbapenemases (known as MBLs), and CVA. The MIC of MEM was the same for both strains (i.e. 0.12 µg ml^−1^), regardless of DPA addition, whereas it decreased to 0.06 µg ml^−1^ upon CVA supplementation (Fig. S2).

**Table 1.** Carbapenem MICs with and without CVA

		MIC (µg ml^−1^)					
Strain		MEM*	IPM	BPM	DOR	FAR	TBP
JBBDAAF-23-N014		0.12	**0.5**	0.25	0.12	2	0.25
	+CVA	0.06	**0.12**	0.25	0.12	2	0.25
JBBDAAF-23-N015		**0.12**	0.25	**0.5**	0.12	2	**0.25**
	+CVA	**0.06**	0.25	**0.25**	0.12	2	**≤ 0.06**

Reductions in MIC values in the presence of CVA are indicated in bold.

*For MEM, the broth microdilution method was performed using manually prepared dilutions, rather than Eiken dry plates (Fig. S2).

DOR, doripenem; FAR, faropenem.

**Fig. 1.**
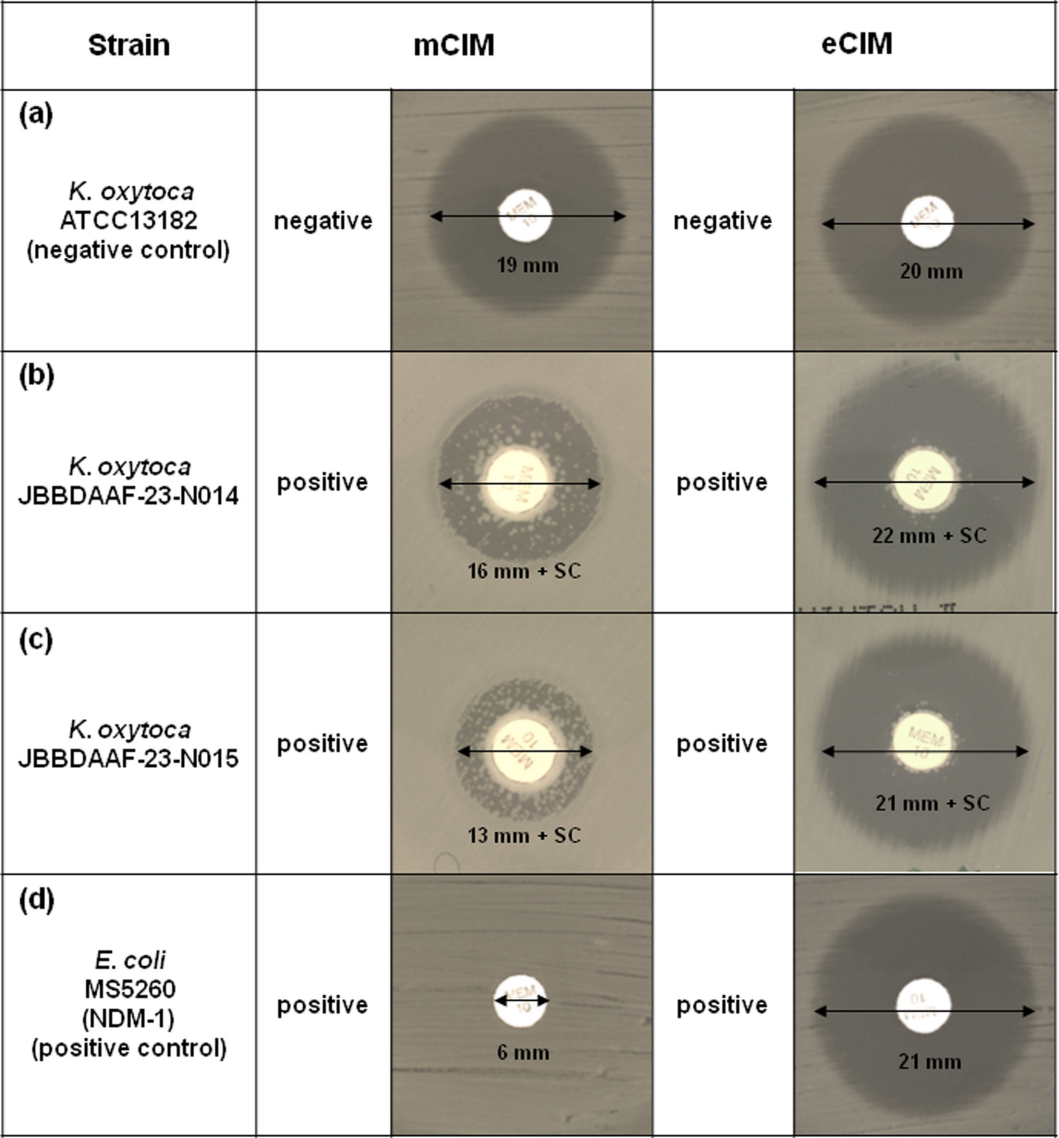
mCIM and eCIM results. The interpretation of the mCIM results was based on the diameter of the inhibition zone. 6–15 mm, positive; 15–18 mm plus satellite colony (SC), positive; 15–18 mm, intermediate; ≥19 mm, negative. eCIM positive: inhibition zone ≥5 mm larger than that of mCIM, indicating MBL. (**a**) *K. oxytoca* (ATCC13182). mCIM: 19 mm, negative; eCIM: 20 mm, negative. (**b**) *K. oxytoca* (JBBDAAF-23-N014). mCIM: 16 mm plus SC, positive; eCIM: 22 mm plus SC, positive. (**c**) *K. oxytoca* (JBBDAAF-23-N015). mCIM: 13 mm plus SC, positive; eCIM: 21 mm plus SC, positive. (**d**) *bla*_NDM-1_-positive *E. coli* strain MS5260. mCIM: 6 mm, positive; eCIM: 21 mm, positive.

Both mCIM and eCIM, the latter being designed to differentiate class B from non-class B carbapenemases using EDTA, a known inhibitor of class B carbapenemases (MBLs), yielded positive results (Fig. 1). In contrast, zCIM, which was designed to enhance low-activity MBL detection through zinc activation, tested negative for both strains: JBBDAAF-23-N014 and JBBDAAF-23-N015 displayed 22 and 21 mm diameter inhibition zones (with satellite colonies), respectively, both exceeding the 20 mm negative result threshold.

**Fig. 2.**
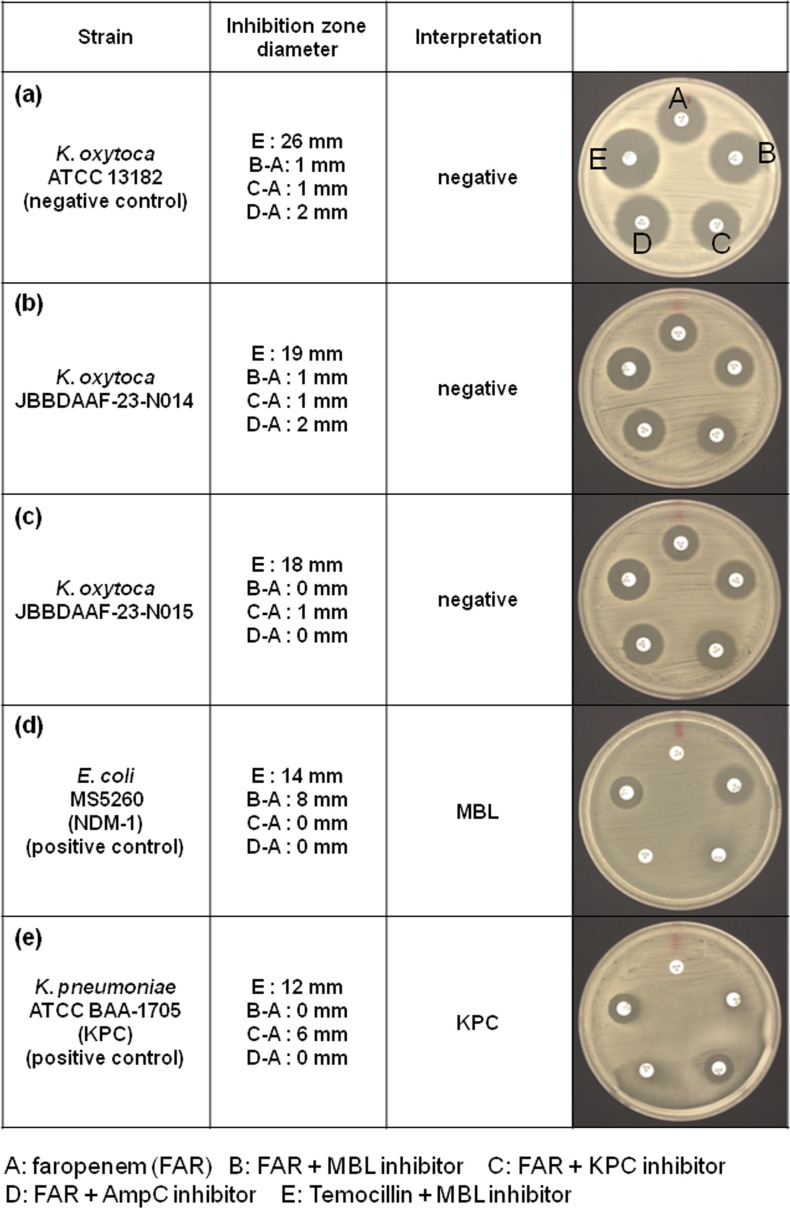
Carbapenemase differentiation discs. Results were interpreted as per the manufacturer’s instructions. MBL: B−A≥5 mm and |C−A| and |D−A|<5 mm; KPC: C−A≥5 mm and |B−A| and |D−A|<5 mm; OXA-48: no synergy observed on A−B, C or D and E≤10 mm; AmpC with porin loss: C−A≥5 mm and D−A≥5 mm and |B−A|<4 mm. Negative: differences among A, B, C and D all ≤2 mm and E >10 mm. (**a**) *K. oxytoca* (ATCC 13182), a negative control displaying E>10 mm with no significant difference in the inhibition zone among A, B, C and D. (**b**) *K. oxytoca* (JBBDAAF-23-N014), negative. (**c**) *K. oxytoca* (JBBDAAF-23-N015), negative. (**d**) *E. coli* MS5260 (*bla*_NDM-1_ positive), a positive control producing MBL exhibiting B and A≥5 mm (due to the MBL inhibitor), C–A<5 mm and D–A<5 mm. (**e**) *K. pneumoniae* ATCC BAA-1705 (*bla*_KPC_ positive), a positive control producing KPC presenting C–A≥5 mm (due to the KPC inhibitor), B–A<5 mm and D–A<5 mm.

Carbapenemase differentiation was performed using the MASTDISCS Combi Carba plus, applying five inhibitor discs to differentiate MBL, KPC, OXA and AmpC *β*-lactamases based on zone diameter comparisons. Neither of the strains (JBBDAAF-23-N014 or JBBDAAF-23-N015) met the criteria for classification as an MBL, KPC or OXA-48 producer, indicating that both strains lacked major carbapenemase production (Fig. 2).

**Fig. 3.**
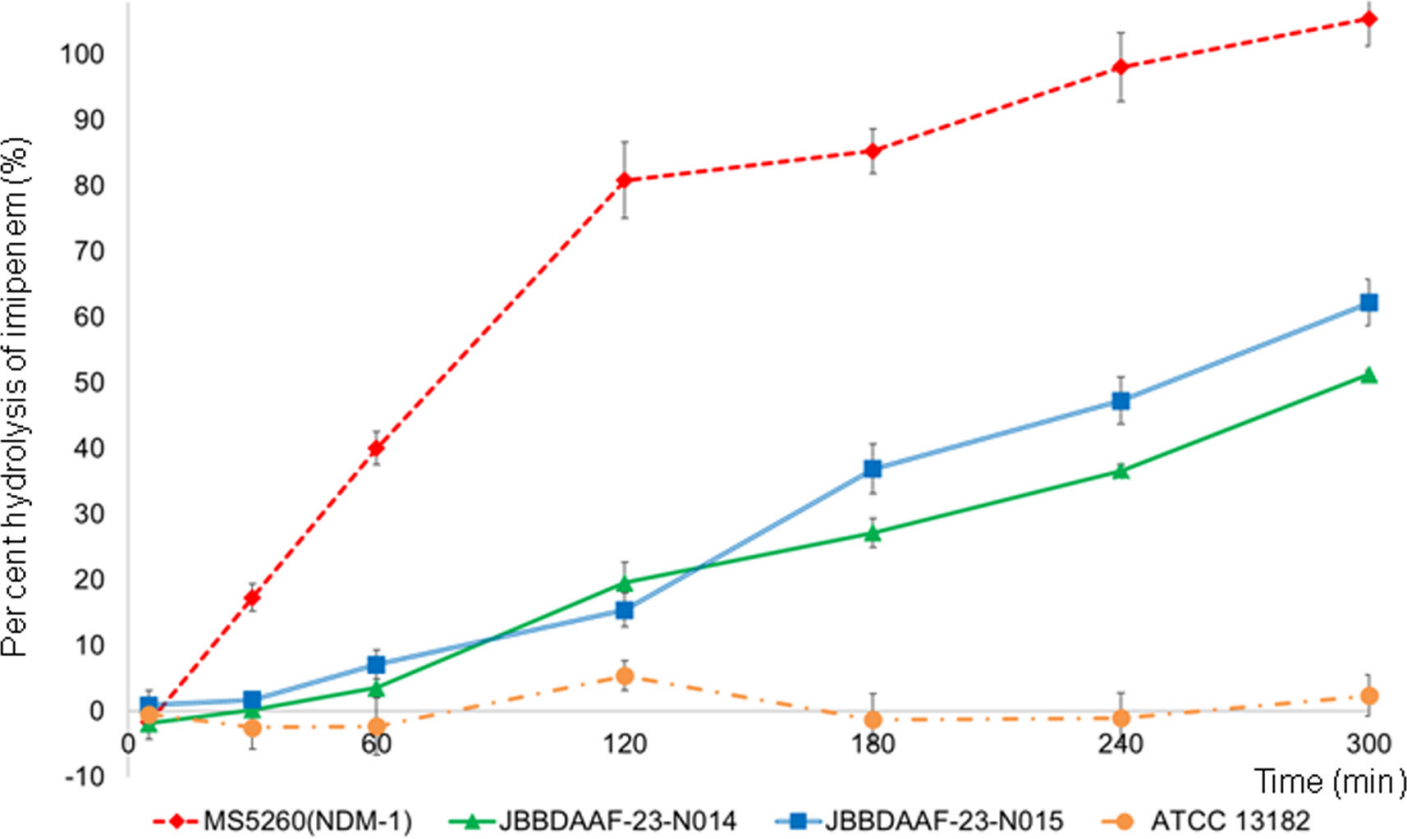
Bacterial hydrolytic activity quantifications. IPM per cent hydrolysis was plotted at 5, 30, 60, 120, 180, 240 and 300 min. The graph represents the mean of three independent measurements for *K. oxytoca* ATCC13182, JBBDAAF-23-N014 and JBBDAAF-23-N015, as well as the *bla*_NDM-1_-positive-producing *E. coli* strain MS5260 plotted in accordance with the labels indicated in the figure. The sd obtained from triplicate experiments are highlighted in the graph.

Furthermore, we conducted a time-course assay to evaluate the carbapenem-hydrolysing activities of the isolated strains. The negative control strain (ATCC 13182) exhibited minimal IPM hydrolysis, whereas the positive control (NDM-1–positive strain MS5260) hydrolysed nearly 100% of the IPM within 240 min. The IPM hydrolysis rates of JBBDAAF-23-N014 and JBBDAAF-23-N015 were ~50–60% at 300 min (Fig. 3), suggesting that the *β*-lactamase OXY-2 produced by these strains could hydrolyse IPM, though less efficiently than NDM-1.

Finally, WGS of the two strains revealed that these isolates did not harbour carbapenemase genes. The only known *β*-lactamase gene identified was *bla*_OXY-2-4_, encoding for a class A *β*-lactamase. No potentially novel *β*-lactamases specific to the isolated strains were identified using HMM profile searches for *β*-lactamases. In addition, a point mutation (GATA[G→A]T), which is known to enhance the expression of this gene [7], was detected in the promoter region of *bla*_OXY-2-4_.

## Discussion

The isolated *K. oxytoca* strains JBBDAAF-23-N014 and JBBDAAF-23-N015 exhibited carbapenem susceptibility with MICs of 0.125 and 0.25 µg ml^−1^ for MEM and IPM, respectively, as determined by the E-test at the reference laboratory. Although these isolates were classified as ‘susceptible’ according to the CLSI breakpoints, their reduced IPM susceptibility, along with the positive results of both the mCIM and eCIM assays, suggested that they might be CPE harbouring carbapenemase genes. However, WGS identified *bla*_OXY-2-4_ as the only known *β*-lactamase gene present, without the detection of any known carbapenemase genes. Furthermore, phenotypic tests, including carbapenemase differentiation disc and MBL detection assays using DPA, yielded negative results, ruling out MBL-type carbapenemase production. These strains were consistently susceptible to cefmetazole and cefepime. Time-course enzyme activity assays assessing carbapenem hydrolysis demonstrated that IPM was gradually degraded by these strains, albeit at a slower rate than that observed in the *bla*_NDM-1_-positive *E. coli* strain. These findings indicate that, although the isolates did not harbour carbapenemase genes, they were capable of hydrolysing carbapenems.

Genomic analysis further revealed that both strains carried a high-expression type promoter mutation (GATA[G→A]T) [7]. *K. oxytoca* clinical isolates produce class A *β*-lactamases (collectively referred to as K1 enzymes) classified into two groups: OXY-1 and OXY-2 [14]. Mutations in the promoter region of *bla*_OXY-1_ or *bla*_OXY-2_ are associated with *β*-lactamase overproduction and resistance to *β*-lactam antimicrobials other than carbapenems [7]. The most frequently observed mutation among 45 *β*-lactam-resistant *K. oxytoca* strains was G→A transition at the fifth nucleotide position, detected in 60 and 72% of the *bla*_OXY-1_- and *bla*_OXY-2_-overproducing strains, respectively, and associated with >100-fold enzyme activity increase [7]. Although rare, a previous case report described an isolate carrying this mutation and exhibiting a MEM MIC of ≥4 µg ml^−1^ [15]. In the two isolates analysed in this study, we observed an MIC reduction of several carbapenems in the presence of CVA, suggesting class A *β*-lactamase activity inhibition and implying that the mCIM positivity in these strains might be attributable to OXY-2 enzyme overproduction, a class A *β*-lactamase intrinsic to *K. oxytoca*. Purified OXY-2-4 reportedly exhibits weak hydrolytic activity against IPM [16,17]. As *bla*_OXY-2_ is typically located on the chromosome, it will likely pose a lower public health concern than carbapenemase genes identified on plasmids, which could disseminate across species, as seen in CPE. However, from a clinical perspective, caution remains warranted owing to the reduced carbapenem antimicrobial susceptibility. Although the two strains carried the same *bla*_OXY-2_ and high-expression type promoter mutation, they differed in the extent of carbapenem MIC reduction following CVA addition (Table 1) and in IPM degradation rates (Fig. 3), suggesting that *bla*_OXY-2_ expression levels may still differ between the two strains.

eCIM positivity further suggests that MEM hydrolysis by OXY-2 was inhibited by EDTA, an MBL inhibitor known to chelate zinc. However, as zinc is not essential for OXY-2 enzymatic activity, the mechanism underlying eCIM positivity remains unclear. Furthermore, the strains tested negative for zinc-dependent carbapenemase production in the zCIM test and were not inhibited by DPA (Fig. S2), supporting the conclusion that these strains are not MBL producers. Taken together, these findings indicate that the strains analysed in this study do not produce carbapenemases but are capable of hydrolysing carbapenems due to OXY-2 overproduction. Strains overproducing OXY enzymes yield positive results in *β*-Carba tests, another carbapenemase screening assay [18]. In the nationwide genomic surveillance conducted by NIID in Japan [4], two other *K. oxytoca* strains with high-expression-type promoter mutations in *bla*_OXY-2_ were identified. However, both tested negative in both mCIM and eCIM assays. These observations suggest that, even among strains carrying identical promoter mutations known to confer high-level expression of *bla*_OXY-2_, mCIM results may vary. In a previous study [15], a *K. oxytoca* strain with a high-expression *bla*_OXY-2_ variant exhibited resistance to MEM but tested negative in mCIM. mCIM negativity of the strain implies that the increased MICs of MEM were due to efflux or reduced drug uptake rather than enzymatic carbapenem degradation. Based on the previous study, we also investigated and identified genes encoding the AcrAB-TolC efflux system (such as *acrA*, *acrB*, *tolC* and their regulatory genes, including *acrR* and *ramA*) as well as porin genes with amino acid substitutions [15], as alternative mechanisms of carbapenem resistance in the whole-genome sequences of the two *K. oxytoca* strains analysed in the present study. Identifying the factors that determine mCIM positivity or negativity among strains with high *bla*_OXY-2_ variant expression represents an important future direction for continued strain collection and surveillance.

Despite the important findings, this study has some limitations. False-positive eCIM results may be attributable to the intrinsic properties of EDTA, such as increased bacterial susceptibility at low concentrations and consequent growth inhibition [19], as suggested by previous reports including an eCIM false-positive case in *Citrobacter sedlakii* [20]. However, the proposed mechanisms were not experimentally validated in the present study, and the true causes therefore remain unclear. Furthermore, in this study, we did not evaluate whether the overexpressed OXY-2, caused by promoter mutations, was directly and exclusively responsible for the hydrolysis of carbapenem antimicrobials. The overexpressed OXY-2 should be investigated in the future using reconstruction experiments by introducing the *bla*_OXY-2_ along with its surrounding sequence, including the promoter, into another strain. Furthermore, elucidating the clinical impact of enzymes capable of hydrolysing carbapenems other than known carbapenemases by collecting additional clinical isolates, assessing the prevalence of isolates with characteristics similar to those observed in the two isolates in the present study and conducting detailed analyses are necessary.

In conclusion, we report that *K. oxytoca* strains lacking known carbapenemase genes can yield positive mCIM results, possibly due to OXY-2 overproduction. These findings highlight the need for caution when interpreting positive mCIM results for *K. oxytoca*, as they do not necessarily indicate carbapenemase production. Furthermore, this study underscores the diagnostic value of combining susceptibility testing with both DPA (an MBL inhibitor) and CVA (a class A *β*-lactamase inhibitor) to differentiate the mechanisms underlying mCIM positivity.

## Supplementary material

10.1099/jmm.0.002139 Uncited Supplementary Material 1.

10.1099/jmm.0.002139 Uncited Table S1.

## Abbreviations

BPM: biapenem
CLSI: Clinical and Laboratory Standards Institute
CPE: carbapenemase-producing *Enterobacterales*
CVA: clavulanic acid
DPA: dipicolinic acid
eCIM: EDTA-carbapenem inactivation method
EUCAST: European Committee on Antimicrobial Susceptibility Testing
HMM: hidden Markov model
IPM: imipenem
MBL: metallo-*β*-lactamase
mCIM: modified carbapenem inactivation method
MEM: meropenem
NIID: National Institute of Infectious Diseases
TBP: tebipenem
WGS: whole-genome sequencing
zCIM: zinc-supplemented carbapenem inactivation method
