# Supplementary material for: Investigation of carbapenem-hydrolysing Klebsiella oxytoca strains lacking detectable carbapenemase genes
Source: J Med Microbiol. 2026 Mar 13;75(3):002139. doi: 10.1099/jmm.0.002139 (PMC12999891; doi:10.1099/jmm.0.002139)
Supplement: Uncited Supplementary Material 1. [file jmm-75-02139-s001.pdf]

Figure S1

Strain	IPM MIC(μg/mL)	MEM MIC(μg/mL)
JBBDAAF-23-N014	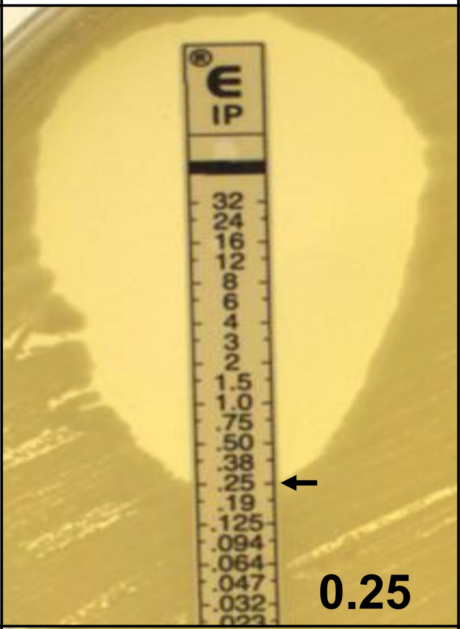<br>0.25	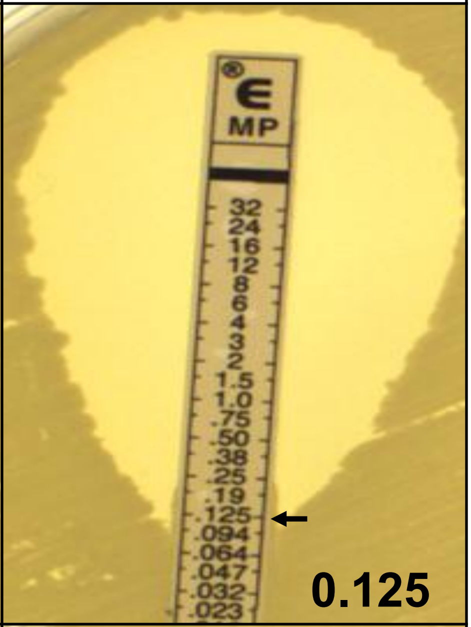<br>0.125
JBBDAAF-23-N015	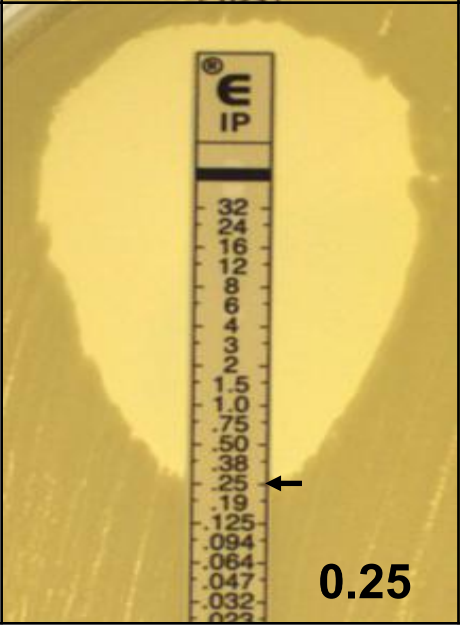<br>0.25	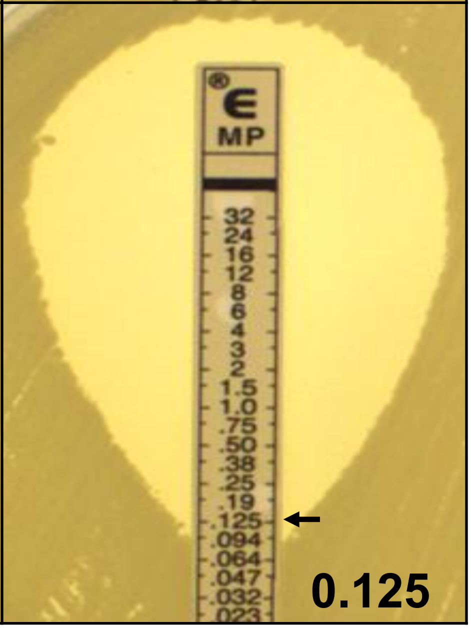<br>0.125

Figure S2

		MIC (µg/mL)
JBBDAAF-23-N014	2 1 0.5 0.25 0.12 0.06 0.03 0015 0.007	
MEM	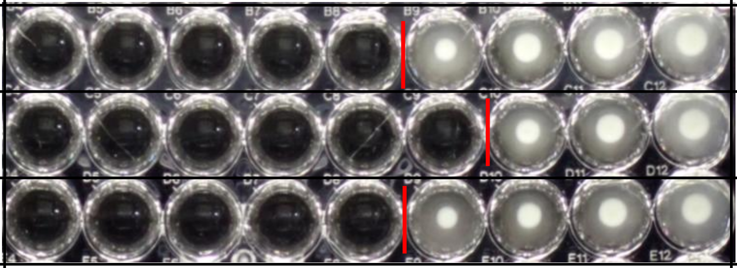	0.12
MEM+CVA		0.06
MEM+DPA		0.12
JBBDAAF-23-N015	2 1 0.5 0.25 0.12 0.06 0.03 0015 0.007	
MEM	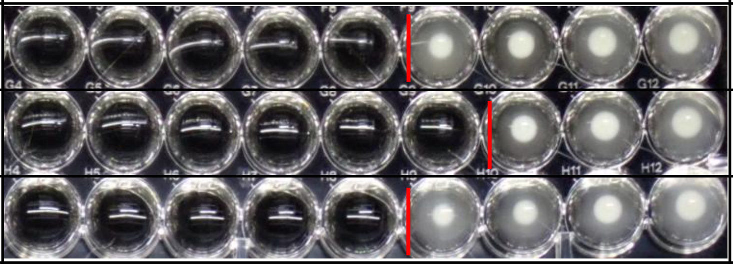	0.12
MEM+CVA		0.06
MEM+DPA		0.12

Supplementary Figures

Figure S1. E-test-measured IPM and MEM MICs (µg/mL)

JBBDAAF-23-N014: IPM 0.25 and MEM 0.125; JBBDAAF-23-N015: IPM 0.25 and MEM 0.125.

Figure S2. MEM MICs (µg/mL) measured using the broth microdilution method

MEM MICs were determined using the broth microdilution method according to the CLSI guidelines. For each strain (i.e., JBBDAAF-23-N014 and JBBDAAF-23-N015), the top, middle, and bottom rows present the results for MEM alone, the middle row shows MEM with clavulanic acid (CVA, 4 µg/mL), and the bottom row shows MEM with dipicolinic acid (DPA, 100 µg/mL).CVA: Clavulanic acid; DPA: Dipicolinic acid; JBBDAAF-23-N014: MEM 0.12, MEM + CVA 0.06, and MEM + DPA 0.12; JBBDAAF-23-N015: MEM 0.12, MEM + CVA 0.06, and MEM + DPA 0.12.
